# Relevance of kinetic interactions and co-formulants for plant protection product liver toxicity in vitro

**DOI:** 10.1007/s00204-025-04071-7

**Published:** 2025-04-28

**Authors:** Yemurai Musengi, Ilinca Suciu, Tewes Tralau, Denise Bloch

**Affiliations:** 1 Department of Pesticides Safety, German Federal Institute for Risk Assessment (BfR), Max-Dohrn-Straße 8-10, 10589 Berlin, Germany; 2 German Federal Institute for Risk Assessment (BfR), Max-Dohrn-Straße 8-10, 10589 Berlin, Germany; 3 Department of Nutritional Toxicology, Institute of Nutritional Science, University of Potsdam, Arthur-Scheunert-Alle 114-116, 14558 Nuthetal, Germany

**Keywords:** Plant protection products risk assessment, Mixture effects, Kinetic interactions, CYP enzymes, Liver steatosis, New approach methodologies (NAMs)

## Abstract

Plant protection product (PPP) risk assessment predominantly focuses on the active substances, at least with respect to the regulatory data requirements. The reason is that for threshold values for the ready-to-use formulations one would need significantly more animal data. Given the high number of formulations, this is not a realistic option, be it for capacity, cost, or animal welfare. Thus, any extension of the data for evaluating formulations should ideally first make maximum use of other approaches. Using an adverse outcome pathway (AOP)-guided approach, we, therefore, investigated the potential mixture effects of two active substances (difenoconazole and mandipropamid) by focusing on the qualitative and quantitative toxicokinetic effects on metabolism. Since difenoconazole is a potential liver steatogen, cytotoxicity and liver triglyceride accumulation in HepaRG cells were used as primary endpoints. In addition, transcriptomics and biochemical analyses were conducted to investigate potential effects on gene expression and inhibition of cytochrome P450 (CYP) enzymes. CA was observed for cytotoxicity of the formulated product-equivalent active substance mixture (1:1); the mixture with a higher concentration of the CYP3A4 inhibitor demonstrated a more than additive effect. Furthermore, modulation of the expression and activity of CYP and steatosis-related nuclear receptors, such as PXR, AhR, and CAR, was observed in the mixtures and the formulated product. Overall, the findings highlight that toxicokinetic interactions between active substances play a vital role in PPP mixture effects and that co-formulants also contribute to this effect. Moreover, this study demonstrates that more than additive effects from kinetic interaction require the enhancer to be within the concentration ranges that potentially saturate CYP enzyme activity. In conclusion, the current findings suggest that a comprehensive PPP risk assessment must consider additive effects between active substances as well as the contribution of co-formulants to adverse effects.

**Supplementary Information:**

The online version contains supplementary material available at 10.1007/s00204-025-04071-7.

## Introduction

Plant protection products (PPPs) are intricate mixtures consisting of one or more active substances and several co-formulants that are designed to maximize biological activity and facilitate the handling, distribution, and delivery of active substances to the intended organisms (Hazen 2000). By design, PPPs contain chemicals that are hazardous to both target and non-targeted organisms, which warrants strict regulations on their use. The toxicity of PPPs is inherently linked to their respective active substances, for which mandatory and extensive mammalian toxicity testing of acute, chronic, and sub-chronic effects is required for approval (EC 2013a). Conversely, the authorization of PPPs containing an approved active substance and co-formulants only requires testing for acute toxicity, skin and eye irritation, and skin sensitization (EC 2013b). In the past, the notion prevailed that co-formulants do not require toxicological evaluation or authorization because they are inert substances that serve supportive functions to improve the efficacy of active substances. In addition, it was and is practically deemed impossible to generate a complete set of in vivo data for each formulation, even if ethics were left aside. Nonetheless, co-formulants can influence the toxicokinetic properties of the active substances and the subsequent toxicity of PPPs (Bloch et al. 2020; Karaca et al. 2023; EC 2009). This influence is unlikely limited to the acute endpoints evaluated for PPP approval (Bloch et al. 2020). Furthermore, several studies have demonstrated that co-formulants can exhibit toxic effects on their own (Song et al. 2011; Chaufan et al. 2014). Therefore, as is commonly observed in complex mixtures, co-formulants can potentially cause or contribute to toxicodynamic interactions or additivity, consequently altering the overall toxicity of the mixture (Backhaus et al. 2013; Mesnage et al. 2014). In addition, multiple active substances in a PPP can result in toxicokinetic and toxicodynamic interactions which may potentially alter PPP toxicity (Kurth et al. 2018).

When multiple active substances co-occur in one PPP, a combined risk assessment is conducted (Stein et al. 2014). This assessment relies on either the concentration addition (CA) concept in the case of mixtures containing active substances with similar modes of action (MoAs), i.e., active substances within the same cumulative assessment group (CAG) (Braeuning et al. 2022), or the independent action (IA) concept in mixtures composed of active substances with dissimilar MoA (Backhaus et al. 2004; Junghans et al. 2005). However, empirical evidence has revealed CA as a pragmatic default approach in risk assessment for mixtures, irrespective of (dis)similar MoAs of its components (Backhaus et al. 2004; Cedergreen 2014; Alarcan et al. 2021). Nevertheless, the respective approach disregards the potential of toxicokinetic interactions of active substances or active substance(s) and co-formulants, which might cause effects that deviate from the typical paradigm of CA, such as synergism (Van Cott et al. 2018; Kurth et al. 2018; Karaca et al. 2023).

In addition to their inherent toxicity, active substances can be substrates, inhibitors, or inducers of CYP enzymes or active transporters. Toxicokinetic interactions may occur when one active substance (toxicological enhancer) inhibits relevant CYP enzymes or active transporters, which increases the bioavailability of another toxic active substance (toxicological driver) and its subsequent adverse effects (Lasch et al. 2021; Karaca et al. 2023; Bloch et al. 2023). However, exposure to the enhancer should be within the inhibitory concentration range (Bloch et al. 2023). Furthermore, previous studies have demonstrated that some pesticides belonging to several chemical classes, such as triazoles, pyrethroids, organochlorines, and organophosphorus pesticides, interact with various influx and efflux drug transporters, such as the efflux pump P-glycoprotein (P-gp) and the influx organic cation transporters (OCTS) (Bain and LeBlanc 1996; Bucher et al. 2013; Mazur et al. 2014; Chedik et al. 2018a). Consequently, the toxicokinetics and overall toxicity of the co-exposed active substances are altered (Guéniche et al. 2019). Co-formulants have long been considered innocuous substances; however, recent studies have linked them to the increased in vitro toxicity of PPPs compared to their active substances (Zahn et al. 2017; Karaca et al. 2021, 2023; Feiertag et al. 2023). These findings have been linked to the enhanced bioavailability of active substances through either increased membrane fluidity, inhibition of active transporters, or inhibition of CYP-mediated metabolism (Woodcock et al. 1992; Karaca et al. 2021; Weinheimer et al. 2016; Rege et al. 2002; Christiansen et al. 2011).

The PPP investigated in this study contains the two active substances, difenoconazole (DIF) and mandipropamid (MDP). It was selected based on potential kinetic interactions between DIF and MDP. Triazole fungicides such as DIF are widely used in agriculture to control fungi in crops by inhibiting fungal CYP51-lanosterol-14α-demethylase, which blocks the synthesis of the fungal cell membrane (Karaca et al. 2023; EFSA 2009). The main target organ for triazoles is the liver, hence, the establishment of the “chronic” CAG for hepatotoxic triazole antifungal agents (EFSA 2009). Triazoles have been reported to induce liver triglyceride accumulation and liver steatosis (Lasch et al. 2021; Heise et al. 2014). Furthermore, triazoles such as DIF are known to interfere with several mammalian CYP enzymes, especially CYP3A4 (Dytrtová et al. 2023; Marx-Stoelting et al. 2020; Lasch et al. 2021). Although MDP has not been investigated as extensively as triazoles, toxicity studies indicate that the liver is the main target organ—only at high concentrations (EFSA 2012). Unlike DIF, MDP does not belong in any CAG. From a regulatory perspective, this indicates that MDP is not considered relevant for cumulative risk assessment either because there are insufficient data on MoA or it exhibits effects that were deemed local or non-adverse (Nielsen et al. 2012). There is currently no data regarding MDP interference with mammalian CYP enzymes.

A putative adverse outcome pathway (AOP) for liver steatosis has been previously established (Mellor et al. 2015; Vinken 2015). Briefly, nuclear receptor (NR) activation, referred to as a molecular initiating event (MIE), triggers key events (KEs) such as NR-associated gene transcription and protein expression, which ultimately induce liver triglyceride accumulation. This accumulation is responsible for cytoplasmic displacement, nucleus distortion, mitochondrial disruption, and endoplasmic reticulum stress (organelle level), which leads to fatty liver cells and ultimately steatosis (Mellor et al. 2015). The MIEs and KEs are conveniently investigated using an established battery of in vitro tests that employs HepaRG cells. HepaRG cells are a useful model for studying hepatic steatosis in vitro*,* as they express important steatosis-related nuclear receptors such as AhR, LXR, PXP, and CAR, as well as exhibit lipid accumulation when treated with known steatotic chemicals. Moreover, HepaRG cells are metabolically competent in vitro models that can further characterize potential toxicokinetic mixture effects in PPPs (Luckert et al. 2018; Knebel et al. 2018; Karaca et al. 2023). Therefore, in this study, differentiated HepaRG cells were used to investigate toxicokinetic and toxicodynamic mixture effects within the selected PPP.

The purpose of this study was to determine whether toxicokinetic interactions between MDP and DIF—potentially dissimilar acting active substances in a PPP—could pose significant concerns regarding the overall toxicity of the PPP. MDP and DIF are contained in a PPP that will be anonymized as “formulated product” throughout the paper. The aim of this study was to investigate the long-term toxicity of a PPP and assess the impact of toxicokinetic interactions between DIF and MDP on the toxicity of the PPP. Toxicological endpoints were selected to address the known effects of DIF on the liver, including cytotoxicity, triglyceride accumulation, CYP phenotyping and inhibition, and changes in the transcriptome of liver cells in vitro.

## Materials and methods

### Product selection and ADMET predictor-based kinetic predictions

Triazole pesticides are extensively investigated and are usually flagged for possible metabolic interactions since they are metabolized by CYP enzymes and also have the ability to both inhibit and induce certain CYP enzymes (Hernández et al. 2012). The adverse effects associated with triazole have been investigated with liver steatosis being of particular interest in this study. Thus, from a large pool of approved PPPs, those containing a triazole and a non-triazole active substance were selected. An initial screening yielded over 100 PPPs; hence, a second screening procedure was conducted to identify possible metabolic interactions. We used ADMET predictor®—a machine learning platform for predicting the absorption, distribution, metabolism, excretion, and toxicity properties of molecules. ADMET predictor® has a defined applicability domain that ranges from 0 to 1, and any compound with a descriptor value below -0.1 and above 1.1 is regarded as out of scope, indicating that it is out of the model’s applicability domain. The out-of-scope results are indicated as either “YES” or “NO” with no predictive confidence, while the in-scope predictions indicate either “YES” or “NO” with predictive confidence in brackets. ADMET predictor® has a risk scoring function for assessing the potential liabilities of a drug molecule. In this study, the scoring function highlighted the risk of high clearance by a specific CYP enzyme as well as possible CYP inhibition liabilities. ADMET predictor® version 11 was used to predict the metabolic properties of triazoles as substrates of the major CYP isoforms involved in xenobiotic metabolism (CYP1A2, CYP2C9, CYP2C19, CYP2D6, and CYP3A4) and the non-triazole active substances as potential inhibitors of the same CYP enzymes. Using the ADMET risk scoring function embedded in ADMET predictor®, we identified the major CYP enzymes responsible for the metabolism of each triazole. Only enzymes with a predictive confidence above 50% were considered. Potential inhibitors of the major five CYP isoforms with predictive confidence above 50% were also considered from the non-triazole active substances list. These predictions were further assessed to determine which PPPs contained a triazole as a substrate for a CYP enzyme that was inhibited by a secondary, non-triazole active substance present in the same PPP.

### Test compounds

The test chemicals difenoconazole (CAS no. 119446–68-3; batch no. BCCJ6184; purity 97.1%) and mandipropamid (CAS no. 374726–62-2; batch no. BCCF9297; purity 98.1%) were obtained from Sigma-Aldrich (Taufkirchen, Germany). The formulated product was purchased from the manufacturer. Both active substances and the formulated product were dissolved in dimethyl sulfoxide (DMSO; CAS no. 67-68-5; batch no. K55014650314) obtained from Sigma-Aldrich (Taufkirchen, Germany) resulting in a final DMSO concentration of 0.5% (v/v) in treatment medium. The active substance and mixture stock solutions’ concentrations were all corrected to the purity of each substance. All stock solutions were stored at – 20 °C. Other chemicals used in this study were obtained from Sigma-Aldrich (Taufkirchen, Germany) or LGC Standards (Wesel, Germany) in the highest available purity.

### Cell culture

Wild-type human hepatoma HepaRG cells obtained from Biopredic International (Saint Grégoire, France) were cultivated for 4 weeks in a Binder cell culture incubator maintained at 37 ºC in a 5% humidified atmosphere with 5% CO_2_ before they were employed in this study. The 4 weeks consisted of 2 weeks of proliferation and 2 weeks of differentiation. In the first 2 weeks, the cells were cultured in William’s Medium E with 2 mM glutamine (PAN-Biotech GmbH; Aidenbach, Germany), supplemented with 10% good forte fetal calf serum (FCS; PAN-Biotech GmbH; Aidenbach, Germany), 100 U/mL penicillin, 100 µg/ml streptomycin (Capricorn Scientific GmbH, Ebsdorfergrund, Germany), 0.05% human insulin (PAN-Biotech GmbH; Aidenbach, Germany), and 50 µM hydrocortisone-hemisuccinate (HHS; Sigma-Aldrich; Taufkirchen, Germany). After 2 weeks of proliferation, HepaRG cell differentiation was initiated by culturing the cells in the same proliferation medium mentioned above but supplemented with 1.7% DMSO for a further 2 weeks. Twenty-four hours prior to treatment with test compounds, the differentiated cells were pre-adapted to treatment medium corresponding to the differentiation medium but supplemented with only 2% FCS and 0.5% DMSO.

Cells were passaged every week for at most five times by aspirating proliferating medium, followed by washing the cells with phosphate-buffered saline (PBS) before incubating them in Dulbecco’s phosphate-buffered saline (Capricorn Scientific GmbH; Ebsdorfergrund, Germany) supplemented with 2 mL trypsin–EDTA (0.05%) at 37 ºC for 2 min. Trypsinization was stopped by adding proliferation medium. Cells were then seeded into 96-well plates at a density of 9000 cells/well or into 24-well plates at a density of 55,000 cells/well.

### Cell viability

The viability of HepaRG cells treated with individual active substances, their mixture as featured in the formulated product, a 1:8 (DIF:MDP) mixture, and the formulated product was analyzed by the water-soluble tetrazolium assay (WST-1; Roche; Berlin, Germany) followed by the neutral red uptake (NRU) assay. Cytotoxicity assays were performed in 96-well plates at a density of 9000 cells per well, and test compounds were incubated for 72 h. Eight different concentrations were investigated in culture medium with a final concentration of 0.5% DMSO, and Triton X-100 (0.1%) served as a positive control. Thirty minutes before the end of incubation, 10 µL of WST-1 reagent was added to each well containing 100 µL medium and further incubated for 30 min. After the 30-min incubation with WST-1, absorbance was measured at 450 nm corrected by a reference at 620 nm (*λ*_exc_ = 450 nm/*λ*_emi_ = 620 nm) using an Infinite M200 PRO plate reader (Tecan; Männedorf, Switzerland). In accordance with Repetto et al. (2008), the NRU assay was performed thereafter onto the same plates. WST-1 medium was aspirated before washing each well with 100 µL PBS. After the wash, 100 µL of neutral red medium was added to each well followed by a 2-h incubation at 37 °C. Afterwards, neutral red medium was aspirated and each well was washed with 100 µL PBS. Subsequently, 150 µL neutral red dye extraction solution (50% ethanol/49% Milli-Q water/1% acetic acid) was added to each well, and the plates were shaken for 10 min at room temperature. The absorbed dye was quantified by fluorescence measurement (*λ*_exc_ = 530 nm/*λ*_emi_ = 645 nm) on an Infinite M200 PRO plate reader (Tecan; Männedorf, Switzerland). Background corrected viability values of both assays were expressed as a percentage of untreated cells. Three independent experiments with three technical replicates were conducted. Means and standard deviations for each treatment were calculated.

### Concentration-additivity modeling

Concentration–response modeling was performed using PROAST software ver. 70 (https://proastweb.rivm.nl/, RIVM, Bilthoven, The Netherlands) in order to examine the nature of the observed mixture effects of the combination of DIF and MDP. The analysis of mixture effects was performed for cytotoxicity. Dose–response curves of the single compounds were used to model a theoretical mixture curve based on the dose addition assumption. In accordance with Kienhuis et al. (2015), the concentration–response data were analyzed by fitting a four-parameter exponential model to the individual substances that showed an effect (MDP, DIF; model: *y* = *a* * [*c* – (*c* – 1) * exp (-bx^*d*^)]). PROAST consecutively shows the analysis based on the exponential and the Hill model. The model with the lowest Akaike Information Criteria (AIC) was selected as the best fitted model. Furthermore, the concentration–response data of the 1:1 and 1:8 mixtures of MDP and DIF were plotted and their locations were compared to the theoretical mixture curve under the assumption of CA (Kienhuis et al. 2015; Karaca et al. 2023). CA can be assumed if the data points of the active substances and their respective mixtures fit well with the theoretical mixture curves. However, a deviation in additivity was assumed when the data points shifted to the left compared to the theoretical mixture curve in case of more than additive/synergistic effect or a shift to the right compared to the theoretical mixture curve for less than additive/antagonistic effect.

### P450-Glo™ screening systems

The P450-Glo™ screening systems (Promega; Madison, Wisconsin, USA) were used in this study to explore the inhibitory properties of MDP on the major CYP isoforms involved in xenobiotic metabolism (CYP1A2, CYP2C9, CYP2C19, CYP2D6, and CYP3A4). HepaRG cells were not utilized in this assay to avoid potential cross reactivity since all CYP enzymes are expressed in this cell line. Therefore, this inhibition study relied on recombinant human CYP enzymes integrated in membrane preparation. The principle of the assay is based on the conversion of a luminogenic substrate (100 µM luciferin-ME for CYP1A2, 100 µM luciferin-H for CYP2C9, 50 µM luciferin-H EGE for CYP2C19, 30 µM luciferin-ME EGE for CYP2D6, and 100 µM luciferin-PPXE for CYP3A4) to luciferin by the respective enzyme. The assay was conducted according to the instructions of the supplier. Briefly, eight non-cytotoxic concentrations of MDP or a CYP selective inhibitor were preincubated for 10 min at 37 ºC together with the luminogenic substrate, potassium phosphate buffer, and luciferin-free water, and the test compounds were added to white 96-well Costar® plates. After the preincubation, 25 µL of NADPH regeneration system (2.6 mM NADP + , 6.6 mM glucose-6-phosphate, 6.6 mM MgCl_2_, and 0.8 U/mL glucose-6-phosphate dehydrogenase) was added to each well to initiate CYP reactions. The plates were briefly shaken and taped before they were incubated at 37 °C for 10–30 min depending on the CYP enzyme under investigation. Luminescence of the luciferin product was initiated by adding 50 µL of the luciferin detection reagent. Subsequently, the plates were briefly shaken and then incubated for 20 min at room temperature in order to stabilize the luminescent signal. The luminescence was measured on the Infinite M200 PRO plate reader (Tecan; Männedorf, Switzerland). The measurements were corrected by subtracting the minus P450 control signals from each measurement and represented as changes in luminescence signals (ΔRLU). The ΔRLU values of MDP were compared with the ΔRLU values of the untreated samples and represented as relative changes in luminescence in relation to the control (%). Three independent experiments with three technical replicates were conducted (n = 3). Means and standard deviations for each treatment were calculated before calculating the IC_50_ values of MDP for each CYP enzyme.

### CYP450 reaction phenotyping with Silensomes™

Silensomes™ CYP phenotyping kits (Lonza; Basel, Switzerland) contain human pooled liver microsomes (HLM) that are chemically and irreversibly inactivated by mechanism-based inhibitor for each specific CYP450 isoenzyme. Each CYP-Silensomes™ has a corresponding control that is produced the same way without inactivation. In this study, CYP1A2-Silensomes™, CYP2C9-Silensomes™, CYP2D6-Silensomes™ and CYP3A4-Silensomes™ were employed to investigate their contribution to DIF metabolism. However, CYP2C19-Silensomes™ were not available for purchasing. The assay was essentially performed according to the instructions of the supplier. In order to investigate the contribution of each enzyme in DIF metabolism, the CYP isoenzymes were supplemented with appropriate co-factors: 1 mM NADPH, 5 mM MgCl_2_, and 0.1 M phosphate buffer (pH 7.4). An experimental volume of 450 µL containing the above-mentioned phase 1 co-factors in the stated final concentrations, Silensomes (final concentration 1 mg/mL) and 1 µM DIF were incubated in a water bath at 37 °C. At 0, 10, 20, 30, and 45 min, 50 µL of the reaction volume was removed and put in a tube containing 50 µL of methanol to stop the CYP reactions. The tubes were allowed to stand at 4 ºC for 1 h to allow protein precipitation to occur. Afterwards, the tubes were centrifuged at 3500 × g for 15 min at 4 ºC before the supernatant was removed and analyzed using LC–MS/MS. The control experiments with the control HLM were performed at the same time as the Silensomes™. The in vitro intrinsic clearance (Cl_int_) for each enzyme was calculated as follows: Cl_int_ (µL/min/mg) = (slope × *V*)/*P*, where the slope is the elimination rate constant (min^−1^) for exponential substrate loss, *V* is the incubation volume (µL), and *P* is the microsomal protein (mg). The fraction metabolized by each CYP enzymes was determined as follows: fm = (1 – (Cl_int_ CYP-Silensomes™/Cl_int_ Control-Silensomes™)) × 100. Three independent experiments were conducted with the same batch of Silensomes™.

### Pesticide quantification in cells and cell culture supernatant

To investigate toxicokinetic mixture effects, pesticide quantification in HepaRG cells and cell culture supernatant was conducted. After seeding and differentiation of HepaRG cells in 24-well plates, cells were subsequently treated with the individual active substances (DIF and MDP), their formulated product-equivalent mixture (1:1), a 1:8 (DIF:MDP) mixture, and the formulated product. HepaRG cells were incubated for 24 h at 37 °C and 5% CO_2_ in a humidified atmosphere with each test compound in treatment medium of 0.5% final DMSO concentration. After incubation, medium was harvested and cells were washed twice with 0.5 mL PBS, which was added to the harvested medium. Cell lysis was performed on ice by adding 0.55 mL of RIPA Lysis and Extraction Buffer (Thermo Fisher Scientific, Waltham, Massachusetts, USA) in each well. The experiment was conducted in duplicates, which were pooled to make a final volume of 3 mL for medium samples and 1.1 ml of the cell samples for each test compound. The Pierce™ BCA Protein Assay Kit (Thermo Fisher Scientific, Waltham, Massachusetts, USA) was utilized to quantify protein in 50 µL of the cell lysates in order to normalize the measured pesticide concentrations in cells to absolute protein quantities.

### Sample preparation

The extraction of the active substance, DIF, from HepaRG cells and medium was done using the Quick, Easy, Cheap, Effective, Rugged, and Safe (QuEChERS) technique with slight deviations from the original method by Anastassiades et al. (2003). Briefly, each of the 1.5 mL of medium samples and 500 µL cell samples was added to 50 mL centrifuge tubes. Up to 5 mL Milli-Q water was added followed by 10 mL acetonitrile. The mixture was shaken vigorously by a vortex mixer for 10 min. Subsequently, one QuEChERS Extract Pouch (Agilent Technologies; Santa Clara, CA, USA) was added to each of the tubes and were immediately shaken by hand for approximately 1 min. Afterwards, the centrifuge tubes were shaken vigorously by a vortex mixer for 10 min and then centrifuged for 5 min at 3000 g and 10 ºC. 800 µL of the supernatant was transferred into autosampler vials containing 200 µL Milli-Q water, and the mixtures were vortexed before analyzing with LC–MS/MS.

### LC–MS/MS analysis

Samples from the CYP450 reaction phenotyping and pesticide quantification were analyzed with an Agilent 1290 Infinity II LC system coupled to Agilent 6475 triple quadrupole LC/MS systems (Agilent Technologies; Heilbronn, Germany). The chromatographic separation was performed on Zorbax Eclipse Plus C18 Rapid Resolution HD (2.1 × 50 mm, 1.8 Micron) (Agilent Technologies; Heilbronn, Germany) using a binary eluent system of 0.1% formic acid in water (mobile phase A) and 0.1% formic acid in methanol (mobile phase B). The gradient conditions were as follows: from 0 to 5 min ramp linearly from 95 to 0% of mobile phase A, then hold for a minute, then ramp over 10 s to initial conditions and hold for another 50 s to re-equilibrate the system. The flow rate was 0.5 mL/min, the injection volume was 3 µL, and the column was maintained at 40° C. Total DIF was detected by multiple reaction monitoring (MRM) in the positive ion mode, quantified by the ion transition *m/z* 406 → 251 at collision energy of 25 V for DIF and *m/z* 412 → 251 at collision energy of 30 V for the internal standard (IS), DIF d6. For confirmation of the analytes, the transitions *m/z* 406 → 188 and 406 → 111 at collision energies of 56 V and 72 V, respectively, for DIF and *m/z* 406 → 188 and 406 → 111 at collision energies of 54 V and 70 V, respectively, for the IS were used. The dwell time was 40 ms for DIF and 20 ms for the IS, while the fragmentation energy was 155 V for DIF and 145 V for the IS. The following source parameters were used: sheath gas flow 11 L/min, gas temperature 350 °C, sheath gas temperature 400 °C, nebulizer 35 psi, gas flow 13 L/min, and cell acceleration voltage (CAV) 5 V. MassHunter software (ver. 12.1) was used for data acquisition and processing, and Graphpad Prism software (ver. 10.1.2) was used for statistical analysis and graphical visualization.

### AOP-based approach

Different elements of the liver steatosis AOP were evaluated in this study. Transcriptomics was employed as a tool to investigate the expression of liver steatosis-related genes that occur either as MIE (genes related to NR activation) or molecular and cellular KEs. In addition to gene expression, lipid accumulation in HepaRG cells was conducted to cover molecular KEs. Considering that upstream MIEs occur earlier than subsequent downstream KEs (Alarcan et al. 2021), we applied different time points (24 h for transcriptomics and 72 h for lipid accumulation) to reflect the temporal relationships between biological events.

### Triglyceride accumulation

The effect of individual active substances, their mixture as featured in the formulated product (1:1), a 1:8 (DIF:MDP) mixture, and the formulated product on triglyceride accumulation was determined using the AdipoRed assay essentially according to the instructions of the supplier (Lonza; Basel, Switzerland). HepaRG cells were treated in 96-well plates for 72 h with eight different concentrations of the above-mentioned test compounds, with the highest concentration of each being the highest non-cytotoxic concentration. The synthetic LXR and FXR agonist, T0901317, was used as a positive control at a concentration of 10 µM. After the 72-h incubation, cells were washed with 200 µL phosphate-buffered saline (PBS), and then 200 µL PBS containing 5 µg/mL Hoechst 33,342 (Thermo Fisher Scientific; Waltham, Massachusetts, USA) was added to each well. Thereafter, 5 µL of the AdipoRed reagent was added to each well, and the plates were incubated at 37 °C for 10 min. Subsequently, the fluorescence of AdipoRed was measured at *λ*_exc_ = 485 nm/*λ*_emi_ = 572 nm, and Hoechst 33,342 was measured at *λ*_exc_ = 350 nm/*λ*_emi_ = 451 nm on the Infinite M200 PRO plate reader (Tecan; Männedorf, Switzerland). AdipoRed signals were normalized to Hoechst 33,342 signals to compensate for variations in cell number. Relative triglyceride levels were referred to untreated cells. Each concentration was measured in three technical replicates. Three independent biological replicates were performed (*n* = 3) and means as well as SD were calculated.

### RNA isolation

After seeding and differentiation of HepaRG cells in 24-well plates, cells were incubated for 24 h with two different non-cytotoxic concentrations of the two individual active substances, their 1:1 and 1:8 mixtures as well as the formulated product (final concentration of 0.5% DMSO). Afterwards, cells were washed twice with 2 mL ice-cold PBS and lysed by adding 350 µL RLT buffer supplemented with 3.5 µL β-mercaptoethanol to each well. RNA isolation was conducted using the RNeasy Mini kit essentially according to the instructions of the supplier (Qiagen; Hilden, Germany). In addition, potential DNA contaminations were digested by incorporating the RNase-Free DNase set (Qiagen; Hilden, Germany) into the RNA isolation protocol. RNA was eluted using 30 µL RNase-free water. The quality and quantity of isolated RNA were examined using a Nanodrop spectrophotometer (NanoDrop 2000; Thermo Fisher Scientific; Waltham, Massachusetts, USA). An A_260_/A_280_ ratio of approximately 2.0 and an A_260_/A_230_ ratio of 2.0 2.2 were accepted as values for pure RNA. RNA integrity was checked by micro-gel electrophoresis using the Agilent RNA 6000 Nano Kit in a Bioanalyzer (Agilent Technologies; Santa Clara, CA, USA). An RNA integrity value of ≥ 8.0 was acceptable.

### Total RNA sequencing

For sequencing analysis, the samples were shipped to Eurofins Genomics Europe Sequencing GmbH (Constance, Germany). Library preparation was performed using Illumina NovaSeq from 150 ng RNA per sample. Sequencing was performed using the Illumina NovaSeq PE 150 sequencing platform. Around 60–100 million paired-end 150 bp reads were produced for each sample. The raw RNA sequencing data are available from Eurofins under accession number NG-A3277. Fastp software (ver. 0.20.0) was used for adapters trimming and data quality assessment. Reads were mapped to the Homo sapiens reference genome UCSC/38/22 and counted per gene ID using STAR (ver. 2.7.10b).

### Transcriptomics statistical analysis

Differential gene expression (DGE) analysis was conducted using the DESeq2 package (Love et al. 2014: https://doi.org/10.1186/s13059-014-0550-8) (version 1.46.0) in R (version 4.4.0), with the integrated development environment RStudio (version 2023.12.1 Build 402). The input data set consisted of ~ 16 k observations, each corresponding to an ENSEMBL gene ID. In cases where multiple ENSEMBL IDs mapped to a single HGNC gene symbol, the read counts were aggregated (summed) and recorded under a single gene symbol entry. Where available, multiple technical replicates were averaged to create a single biological replicate entry for each condition. For exploratory analysis, the normalized expression counts were transformed using the variance-stabilizing transformation (vst). Principal component analysis (PCA) and Pearson correlation plots were generated to assess clustering patterns and identify any outliers. All data points were retained for further analysis. To account for overdispersion in RNA-Seq data (where variance exceeds the mean), a negative binomial model was fitted to the count data. Differential expression was assessed using the Wald test, which tested the effect of treatment relative to DMSO controls, while controlling for biological variation. To account for multiple comparisons, the false-discovery rate (FDR) was controlled using the Benjamini–Hochberg correction (*α* = 0.05). Genes were considered significantly differentially expressed if the adjusted p-value (FDR) was less than 0.05 and the absolute fold change was at least 2.

### Statistical analysis

Graphpad Prism software (ver. 10.1.2) was used for statistical analysis, graphical visualization, curve fitting of the data as well as EC_50_/IC_50_ computation. A linear mixed-effects ANOVA (*α* = 0.05) described by Pinheiro and Bates (2000) followed by a post-hoc Dunnett test for multiple comparisons of treatment groups versus the control was used for statistical analysis of data from cytotoxicity assay, AdipoRed assay, CYP450 inhibition assay as well as mixture effects. R studio (ver. 4.4.1) was used to determine the effective concentration at relative triglyceride induction fold of 1.5 for each test compound.

## Results

### ADMET predictor-based kinetic predictions

Based on the approved active substances in Germany, nine triazoles were identified (https://ec.europa.eu/food/plant/pesticides/eu-pesticides-database/start/screen/active-substances; Accessed: September 2023). Based on these, about 210 PPPs were selected as a starting point for this study (https://psm-zulassung.bvl.bund.de/psm/jsp/; Accessed: September 2023). PPPs with no dermal absorption data, more than two active substances, active substances in the same CAG, a second active substance that is not an inhibitor of the relevant CYP enzymes for triazole metabolism as well as a triazole substrate prediction confidence of below 50 were excluded. Furthermore, PPPs that contained triazoles that are not in any CAG group were excluded. This screening yielded one PPP, which was then used for further mixture effect investigation. Table 1 summarizes the results yielded from ADMET predictor® for this PPP.

**Table 1:** Summary of the CYP substrate/inhibitor predictions of DIF and MDP, the active substances of the formulated product generated by ADMET predictor® ver.11.0

CYP enzyme	Inhibitor		Substrate	
	MDP	DIF	MDP	DIF
CYP1A2	Yes (95%)	Yes (64%)	No (59%)	Yes (69%)
CYP2C9	Yes (89%)	Yes (89%)	Yes (37%)	Yes (33%)
CYP2C19	No	Yes (91%)	No (68%)	Yes (81%)
CYP2D6	Yes (58%)	Yes (58%)	No	No (79%)
CYP3A4	Yes	Yes (78%)	Yes (86%)	Yes (98%)

### Cell viability

**Fig. 1:**
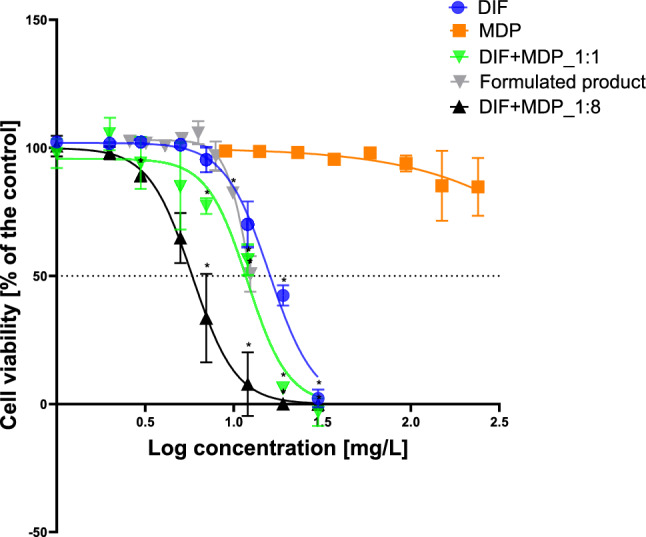
Results of NRU cytotoxicity test in HepaRG cells after 72-h exposure to increasing concentrations of DIF, MDP, a mixture of DIF and MDP as featured in the formulated product (1:1), a 1:8 (DIF:MDP) mixture and the formulated product. The data are presented as the percentage of viable cells related to the solvent control. The data represent mean values ± SD of n = 3 biological replicates, each performed in three technical replicates. An asterisk (*) indicates statistical significance with *α* ≤ 0.05 after a linear mixed-effects ANOVA statistical analysis followed by a post-hoc Dunnet test

Cytotoxicity was the first metric used to compare the overall toxicity of the formulated product and its active substances (DIF and MDP) individually and in combination. The cell viability data for all tested compounds indicated a dose–response relationship with increasing concentrations, except for MDP (Fig. 1). Table 2 provides a summary of the EC_50_ values derived from the best fit values of the dose–response curves generated from the NRU assay.

**Table 2:** EC_50_ values for cytotoxicity derived from best fit values of a non-linear dose–response curve of all treatment substances on HepaRG cells after 72-h incubation

	DIF	MDP	DIF + MDP_1:1	DIF + MDP_1:8	Formulated product
EC_50_ (mg/L)	15.9	1224	12.0	5.84	12.3
95% CI	14.1 to 17.0	421 to 419,341	9.99 to 13.8	5.27 to 6.39	12.0 to 12.7

Both mixtures of DIF and MDP exhibited significant cytotoxic effects at lower concentrations compared to the individual active substances. However, the 1:8 (DIF:MDP) mixture showed the highest toxicity to HepaRG cells, indicative of the effect of CYP enzyme inhibition properties of MDP on the overall toxicity of the mixture. The ready formulated product showed more cytotoxic effects compared to the individual active substances. However, in contrast to the formulated product-equivalent mixture of DIF and MDP (1:1), the formulated product did not exhibit more cytotoxic effects up to the maximum tested concentration. MDP was not cytotoxic in the tested concentration range. Furthermore, the WST-1 assay used as a confirmatory cytotoxicity test yielded largely comparable findings (Fig. S1.)

### Concentration addition modeling

**Fig. 2:**
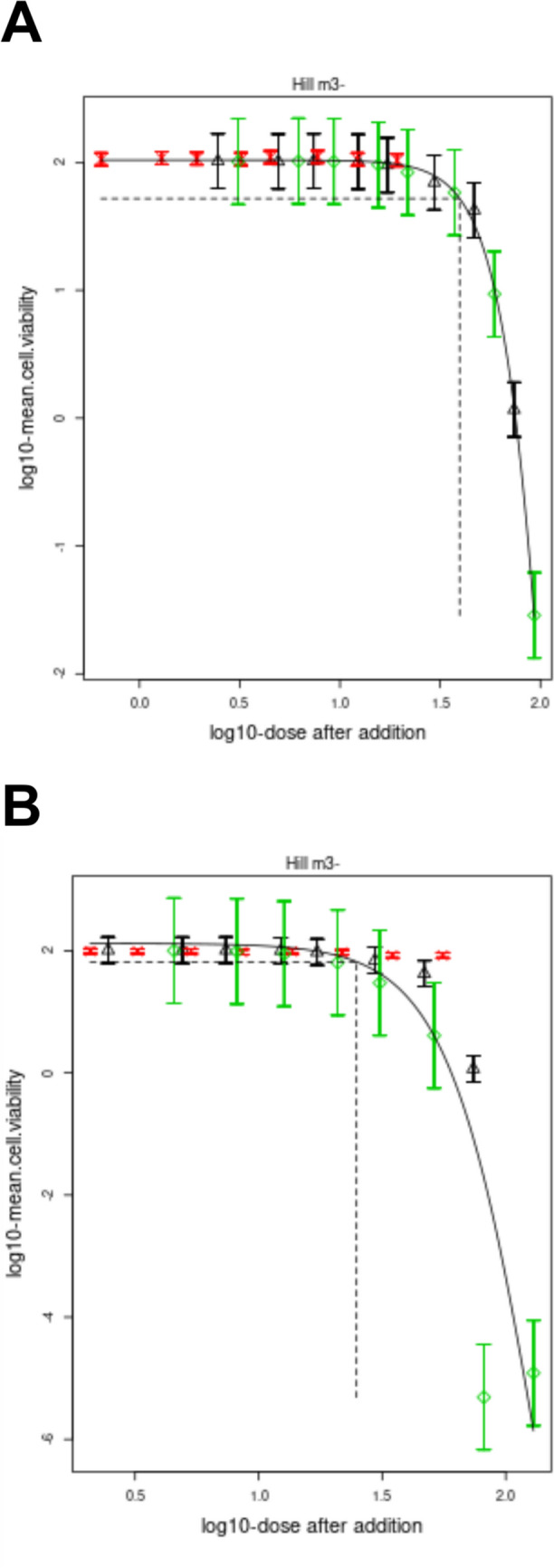
Concentration–response modeling of the cytotoxicity results on HepaRG cells. Shown are the comparison of the additive models (black lines) with the experimental data for DIF (black upward triangles), MDP (red cross) and **A** 1:1 mixture of DIF and MDP (green diamond) or **B** 1:8 mixture of DIF and MDP. CA modeling was performed using the web version PROAST software ver. 70.1

To further validate any observed mixture effects, concentration- or over-additivity, all the toxicity data referring to the formulated product were subsequently subjected to CA modeling (Fig. 2). As highlighted in Fig. 2A, the binary mixture of DIF and MDP, as featured in the ready formulated product, fitted well to the modeled effect curve, indicating that the mixture follows an additive behavior. However, this was altered when the ratio of DIF to MDP was increased to 1:8 (Fig. 2B). This 1:8 binary mixture was located slightly to the left of the modeled effect curve, especially for the highest concentration, indicating a more than additive effect.

### P450-Glo™ inhibition assay

**Fig. 3:**
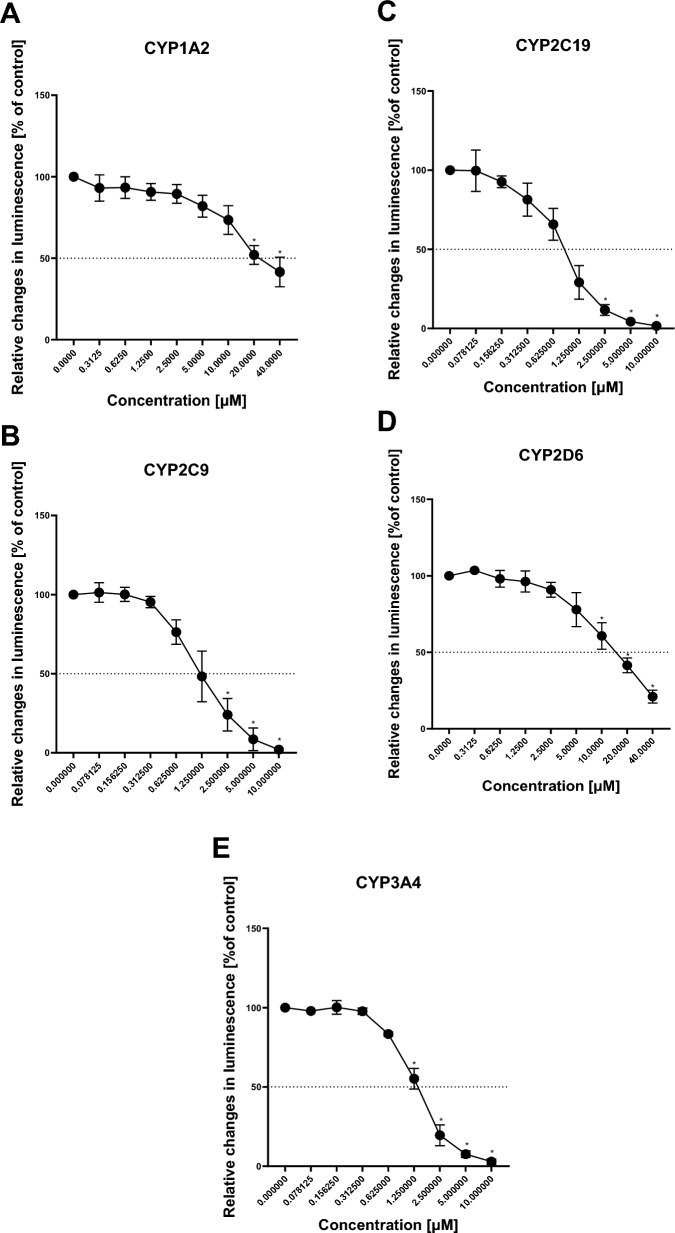
CYP enzyme activity for CYP1A2 (**A**), CYP2C9 (**B**), CYP2C19 (**C**), CYP2D6 (**D**), and CYP3A4 (**E**) presented as relative changes in luminescence compared to basal activity (solvent control) and corrected for the non-enzymatic control following exposure to increasing concentrations of MDP. Curve modeling and IC_50_ calculations were performed using a four-parameter model: Y = Bottom + (Top–Bottom)/(1 + 10 ^ ((LogIC50—X) *Hillslope)) in Graphpad Prism software. The data represent mean values ± SD of n = 3 biological replicates, each performed in three technical replicates. Statistical analysis was done by a linear mixed-effects ANOVA (α ≤ 0.05) followed by a post-hoc Dunnett test. An asterisk (*) indicates statistical significance compared to solvent control

The inhibitory properties of MDP were assessed as a potential contributing factor to any of the observed mixture effects using the P450-Glo™ screening systems. As depicted in Fig. 3, MDP exhibited concentration-dependent inhibition of CYP1A2, CYP2C9, CYP2C19, CYP2D6, and CYP3A4 with significant inhibition at higher concentrations. Although MDP was found to be an effective inhibitor for the above-mentioned CYP enzymes, Table 3 shows that it is a more potent inhibitor of CYP2C19 indicated by the lowest half-maximal inhibitory concentration (IC_50_) of 0.824 µM (0.340 mg/L). In addition, MDP appeared to be a strong inhibitor of CYP3A4, a key enzyme in DIF metabolism with an IC_50_ of 1.32 µM (0.544 mg/L). As evidenced by the highest IC_50_ of 28.7 µM (11.8 mg/L), MDP shows the lowest potency for CYP1A2, another key enzyme in DIF metabolism based on ADMET predictor®. The inhibition potency of MDP towards CYP enzymes ranks as CYP2C19 > CYP2C9 > CYP3A4 > CYP2D6 > CYP1A2 with IC_50_ values summarized in Table 3.

**Table 3:** IC_50_ values for each CYP enzyme derived from data of three independent P450-Glo™ CYP enzymes’ inhibition assay with MDP

CYP enzyme	IC_50_ [µM]	IC_50_ [mg/L]	Control inhibitor
CYP1A2	28.7	11.8	α-Naphthoflavone (1 µM)
CYP2C9	1.18	0.488	Sulfaphenazole (10 µM)
CYP2C19	0.824	0.340	Troglitazone (10 µM)
CYP2D6	18.1	7.47	Quinidine (1 µM)
CYP3A4	1.32	0.544	Ketoconazole (5 µM)

Respective control inhibitors and their concentrations are also summarized

### CYP450 reaction phenotyping

**Fig. 4:**
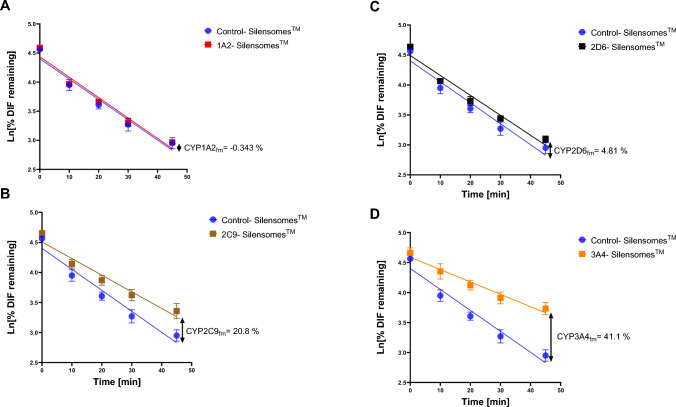
DIF kinetics with CYP1A2-Silensomes™ (A), CYP2C9-Silensomes™ (B), CYP2D6-Silensomes™(C), and CYP3A4-Silensomes™ (D). 1.1 µM DIF was incubated with microsomes and co-factors for different time points. Data represent mean values ± SD from three biological replicates conducted with the same batch of Silensomes™

According to Wetmore et al. (2014), DIF is mainly metabolized by CYP3A4. Furthermore, ADMET predictor® predicted CYP1A2, CYP2C9, CYP2C19, and CYP3A4 to be involved in DIF metabolism. For this reason, CYP450 reaction phenotyping using Silensomes™ was conducted to determine the contribution of the major CYP isoforms involved in xenobiotic metabolism. The depletion of DIF over time is shown in Fig. 4. As depicted by Fig. 4A, when DIF was incubated with control-Silensomes™ and CYP1A2-Silensomes™, about 20% of the chemical was remaining after a 45-min incubation. The estimated CL_int_ of control-Silensomes™ and CYP1A2-Silensomes™ were 15.7 µL × min^−1^ × mg^−1^ and 15.8 µL × min^−1^ × mg^−1^ respectively. From this, the CYP1A2 *fm* was evaluated to be ≈ 0%, which indicates that CYP1A2 is not involved in DIF metabolism. Conversely, Fig. 4B shows that CYP2C9 contributes about 21.8% to DIF metabolism with an estimated in vitro intrinsic clearance (CL_int_) of 12.5 µL × min^−1^ × mg^−1^. CYP2D6 has about 4.81% contribution to DIF metabolism as shown in Fig. 4C by only a slight deviation from the control-Silensomes™ curve. Contrary, for CYP3A4-Silensomes™, DIF (Fig. 4D) depletion was slower compared to the control-Silensomes™. Furthermore, about two times the amount of DIF was remaining (40%) after 45 min compared to that of control-Silensomes™. In vitro intrinsic clearance (CL_int_) was estimated to be 9.27 µL x min^−1^ × mg^−1^ for CYP3A4-Silensomes™. Consequently, it was estimated that CYP3A4 contributed about 41.1% to DIF metabolism. The findings indicate that CYP3A4 is the key CYP enzyme in DIF metabolism with the highest *fm* of 41.1%.

### Quantification of DIF in cells and cell culture supernatant

**Fig. 5:**
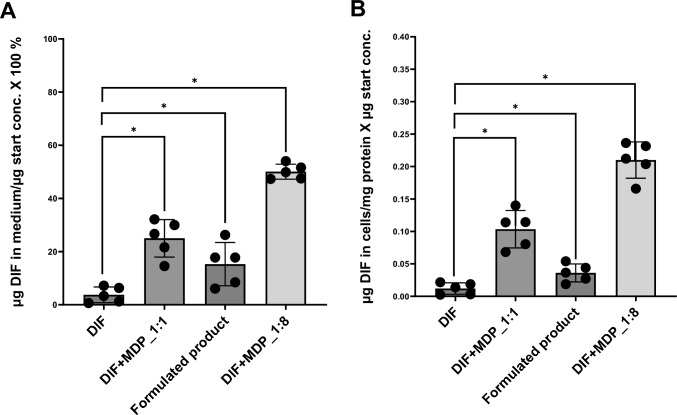
Quantification of DIF after 24-h incubation with DIF, the formulated product-equivalent mixture (1:1), 1:8 (DIF:MDP) mixture, and the formulated product in **A** HepaRG cell medium presented as µg DIF in medium/µg start concentration × 100% and **B** HepaRG cells presented as µg DIF in cells/mg protein x µg start concentration. Data represent mean values ± SD from three biological by two technical replicates. Statistical analysis was done by a linear mixed-effects ANOVA (*α* ≤ 0.05) followed by a post-hoc Dunnett test. An asterisk (*) indicates statistical significance compared to DIF treatment

As elaborated in the CYP inhibition and reaction phenotyping assay above, MDP is an inhibitor of CYP1A2 and CYP3A4, the latter being a relevant enzyme for DIF metabolism. Accordingly, the hypothesis of this study is that MDP decreases the metabolism of DIF leading to an increase in DIF intracellular and extracellular concentration in the mixture and the formulated product treatment relative to DIF treatment, resulting in increased toxicity. We, therefore, investigated the extent to which MDP-mediated enzyme inhibition would increase DIF bioavailability in the formulated product by quantification of DIF in cells and cell culture medium after treatment with DIF, the formulated product-equivalent mixture (1:1), 1:8 (DIF:MDP) mixture and the formulated product (Fig. 5). The results show that almost 4% of DIF remained in the cell culture medium after 24-h treatment of HepaRG cells with DIF alone, whereas in the formulated product-equivalent mixture (1:1), approximately 25% of DIF remained. This suggests that MDP significantly inhibits the metabolism of DIF (Fig. 5A). In contrast to the mixture, the formulated product showed a lower concentration of DIF remaining in the cell culture medium (15.3%), suggesting toxicokinetic interactions that go beyond those exhibited by MDP and DIF. The results for the 1:8 (DIF:MDP) mixture (50%) in Fig. 5A demonstrated that increasing the concentration of the inhibitor further increased the concentration of the remaining DIF in HepaRG cell culture medium. The same pattern was observed for intracellular concentrations of DIF where relative to the single compound treatment, the cells treated with the 1:8 (DIF:MDP) mixture showed the highest concentration of remaining DIF followed by the formulated product-equivalent mixture (1:1) and then the formulated product (Fig. 5B).

### Triglyceride accumulation

**Fig. 6:**
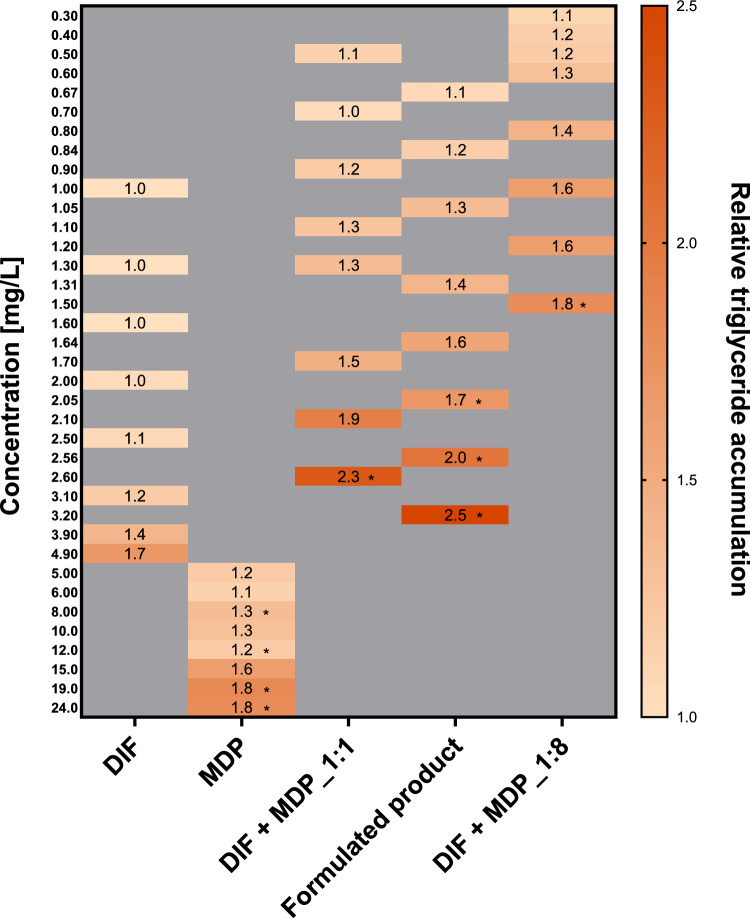
Results of the AdipoRed assay after 72 h of exposure to sub-cytotoxic concentrations of DIF, MDP, their formulated product-equivalent mixture (1:1), a 1:8 (DIF:MDP) mixture, and the formulated product (expressed as the active substances’ concentration within the formulated product). Data are presented as relative values of triglyceride accumulation related to the medium control (0.5% DMSO). The data represent mean values ± SD of *n* = 3 biological replicates, each performed in three technical replicates. Statistical analysis was done by a linear mixed-effects ANOVA (*α* ≤ 0.05) followed by a post-hoc Dunnett test. An asterisk (*) indicates statistical significance compared to solvent control

AdipoRed assay was used to evaluate liver triglyceride accumulation, a crucial functional endpoint of hepatic steatosis. Prior to staining with AdipoRed dye to determine triglyceride content, HepaRG cells were incubated for 72 h with the test compounds. As depicted in Fig. 6, the individual active substances, the mixtures, and the formulated product increased triglyceride content in HepaRG cells in a concentration-dependent manner. However, Fig. 6 signifies a shift to the left for the mixtures and the formulated product curves relative to those of the individual substances, indicating that the mixtures and the formulated product are more toxic than the individual active substances. As summarized in Table 4, while DIF exhibited steatotic effects at concentration as low as 4.21 mg/L, MDP did not induce any significant triglyceride accumulation up to 14.3 mg/L. When combined with MDP, the triglyceride content in the cells increased already at low DIF concentrations at which no steatotic effect was observed. The formulated product induced triglyceride accumulation at concentrations lower than the individual active substances and the formulated product-equivalent mixture (1:1). In contrast to the other test compounds, the mixture with eight times the amount of the inhibitor, MDP induced triglyceride accumulation at the lowest concentration of 0.930 mg/L, as highlighted in Table 4.

**Table 4:** Estimated effective concentration at 1.5 relative triglyceride induction fold (EC_1.5-fold_) derived from best fit values of a non-linear dose–response curve of all treatment substances on HepaRG cells after 72-h incubation

	DIF	MDP	DIF + MDP_1:1	DIF + MDP_1:8	Formulated product
EC_1.5-fold_ (mg/L)	4.21	14.4	1.55	0.930	1.54
95% CI	3.07–5.36	11.7–17.1	– 1.23 to 4.34	– 0.43 to 2.29	– 1.89 to 4.96

### Transcriptome analysis

**Fig. 7:**
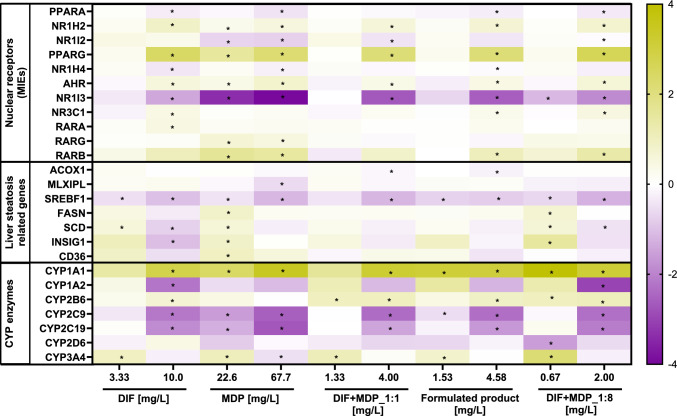
RNA expression patterns of HepaRG cells after 24-h exposure to sub-cytotoxic concentrations of DIF, MDP, and DIF + MDP in ratios of 1:1 and 1:8 and the formulated product (expressed as the active substances’ concentration within the formulated product). The results are presented as log 2 values of fold change in comparison to solvent control. Up- and downregulations were considered relevant at ≤ 0.5- and ≥ twofold change in gene expression. The data represent mean values ± SD of n = 3 biological replicates, each performed in one technical replicate. Statistical analysis was performed for each gene to compare treatment groups and control generating p-values for each gene. The p-values were corrected by determining false-discovery rates (FDR) using the Benjamin–Hochberg method. An asterisk (*) indicates significant differentially expressed genes with adjusted p-value ≤ 0.05. The full data set can be found in Supplementary Table 2

The relative expression of genes involved in phase I metabolism and those included in the liver steatosis AOP network was investigated to better understand the molecular mechanism leading to additive and over-additive adverse effects on a cellular level. Transcriptome analysis shows a similar gene expression profile after 24-h incubation with high sub-cytotoxic concentrations of DIF, MDP, and DIF + MDP in ratios of 1:1 and 1:8, and the formulated product (Fig. 7). As shown in Fig. 7, the high MDP concentration showed a downregulation of all CYP enzymes except CYP1A1 (≈ 11-fold upregulation) and CYP2B6, which remained unchanged. The gene expression profiles of CYP1A1 and CYP2B6 indicate no effect on CAR but an upregulation on AHR. The downregulation of the five major CYP enzymes is in line with the results from the inhibitory assay where MDP likewise inhibited all the five CYP enzymes with the strongest effect on CYP2C9 and CYP2C19. Interestingly, the low MDP concentration slightly upregulated CYP3A4 (≈ twofold) with other CYPs showing the same pattern as the high concentration. The high MDP concentration showed a significant downregulation of SREBF1 (≈ 0.45-fold), while little to no effect with a weak downregulation or upregulation at best was observed with respect to other steatosis-related genes. Both low and high concentrations of MDP caused a strong downregulation of NR1I3 (CAR) with ≈ 0.1-fold and ≈ 0.06-fold, respectively. PPARG and RARB were significantly upregulated by high and low concentrations of MDP, whereas NR1H2, AHR, NR3C1, RARA, and RARG were weakly upregulated. Both concentrations of DIF showed a similar gene expression profile to MDP, with the high concentration efficiently upregulating CYP1A1 (≈ sevenfold), RARB (≈ twofold), and PPARG (≈ 5.3-fold), while significantly downregulating CYP1A2 (≈ 0.2-fold), CYP2C9 (≈ 0.2-fold), CYP2C19 (≈ 0.2-fold), and NR1I3 (≈ 0.37-fold). The high DIF concentration showed a significant downregulation of SREBF1 (≈ 0.50-fold) and INSIG1 (≈ 0.48-fold), while the low DIF concentration exhibited an upregulation of INSIG1 (≈ 2.6-fold). Other steatosis-related genes except MLXIPL were found to be weakly downregulated in the high concentration, while the low concentration weakly upregulated these genes. As depicted in Fig. 7, the mixtures of DIF and MDP and the formulated product followed the same gene expression profile as the individual active substances. At high concentrations, CYP2C9 and CYP2C19 were significantly downregulated, while CYP3A4 showed no effect with regard to the formulated product and weak downregulation by both mixtures. Contrastingly, CYP1A1 was significantly upregulated by both mixtures and the formulated product indicating an effect on AHR. The high concentration of DIF + MDP (1:8) mixture showed the strongest upregulation of CYP2B6 (≈ twofold). Although the DIF + MDP (1:8) mixture caused a significant downregulation of CYP2D6 (≈ 0.34-fold) and weak upregulation to no effect on CYP2C19 at low concentration, the DIF + MDP (1:1) mixture and the formulated product showed a weak downregulation for the two CYPs. The mixtures of DIF and MDP significantly upregulated CYP3A4 compared to the low concentrations of individual substances. This indicates that the presence of the DIF and MDP in mixture significantly modulates the gene expression of this CYP enzyme. However, the upregulation observed in the formulated product was lower compared to both mixtures (≈ twofold and ≈ 4.5-fold) and MDP (≈ twofold), which is indicative of potential modulation of CYP enzyme gene expression by co-formulants in the formulated product. The high concentrations of both mixtures showed a significant downregulation of SREBF1 (≈ 0.40-fold), while the other steatosis-related genes exhibited little to no effect with a weak downregulation or upregulation at best. At low concentration, the DIF + MDP (1:8) mixture showed an upregulation of INSIG1 (≈ 2.8-fold). Conversely, both concentrations of the formulated product caused weak modulation of steatosis-related genes. PPARG and RARB were significantly upregulated by the DIF + MDP (1:8) mixture and the formulated product at high concentrations, while both mixtures and the formulated product significantly downregulated NR1I3 (CAR).

## Discussion

This study evaluated the impact of toxicokinetic interactions between two active substances. The selected PPP consisted of MDP, which is not associated with any CAG and DIF, a steatotic fungicidal triazole that is widely used in PPPs and associated with the CAG for the target organ liver (EFSA 2009). Based on DIF’s MoA, a liver steatosis AOP was employed as a conceptual framework to evaluate the mixture effects on a sequential cascade of events with various endpoints covering several layers of biological significance rather than a single endpoint. Our findings demonstrate that the nature and intensity of the observed mixture effects in the formulated product were influenced by the ratio of the active substances, particularly the quantity of the toxicological enhancer. In ratios corresponding to the formulated product, DIF and MDP displayed additive effects, while they resulted in more than additive effect at higher concentrations of the 1:8 mixture. Here, we discuss the impact of the two active substances and co-formulants on the toxicokinetic and steatosis-related dynamic effects in vitro.

### Toxicokinetics interactions

The in vitro CYP interaction between MDP and DIF was predicted by ADMET predictor®, which identified MDP as an inhibitor of all investigated CYPs except CYP2C19 and DIF as a substrate of all CYPs except CYP2D6 (Table 1).

In contrast to other predictive tools, Zhai et al. (2023) demonstrated that for metabolism, ADMET predictor® had the poorest specificity and the highest sensitivity for identifying true inhibitors, which tend to have a higher false positive rate. In silico tools such as ADMET predictor® are largely dependent on large experimental databases for better coverage and performance (Tyzack and Kirchmair 2018; Esaki and Ikeda 2023). However, data availability is often a limiting factor, especially for non-pharmaceutical substances. Thus, model development for these chemicals depends on pharmaceutical data or data from open databases, which require expert curation (Esaki and Ikeda 2023). Although ADMET predictor® incorporates advanced machine-learning algorithms (Sohlenius-Sternbeck and Terelius 2021; Swanson et al. 2024), its predictions are most reliable for compounds similar to those in its training sets. Binary in silico predictions may not be relevant to concentrations employed in in vitro experiments where solubility, distribution in cells, and interactions with biological components are some of the variables that affect actual responses (Garcia de Lomana et al. 2021). Furthermore, the “yes” or “no” outcome provided by ADMET predictor® is entirely qualitative. The model could not quantitatively predict the role of MDP in influencing the expression of each enzyme or the contribution of each enzyme to the overall metabolism of DIF. Therefore, while in silico tools are useful for screening (Myatt et al. 2018; Belfield et al. 2021), coupling with in vitro tools is necessary to fully comprehend the relevance of enzyme interaction by either directly evaluating CYP interaction or indirectly accounting for interaction in toxicity assays.

In vitro experiments revealed that MDP inhibits all major CYP450 enzymes involved in xenobiotic metabolism (Fig. 3), with CYP2C9, CYP2C19, and CYP3A4 showing the most potent inhibition, thereby altering cellular xenobiotic-metabolizing capacity. To the best of our knowledge, there have been no previous studies on the interaction between MDP and any CYP enzymes.

Our results showed that DIF is mainly metabolized by CYP2C9 (≈ 20%) and CYP3A4 (≈ 40%) (Fig. 4). As a triazole, DIF has demonstrated the importance of CYP450 enzymes with regard to potential toxicity and detoxification. Previous studies have reported that DIF is preferentially metabolized by CYP3A4 (Wetmore et al. 2014; Lasch et al. 2021), which is consistent with the CYP450 reaction phenotyping results (Fig. 4). Furthermore, Wetmore et al. (2014) demonstrated that CYP3A4 (*fm* = 43.3%), CYP2C9 (*fm* = 5.41%), and UGT1A4 (*fm* = 4.55%) were the main contributors to the clearance of DIF in healthy human adults.

Inhibition of xenobiotic-metabolizing enzymes by one substance in the mixture can increase the metabolism of other compounds, thereby altering the concentration at the target site (Hernández et al. 2012; Bloch et al. 2023). By disrupting phase I metabolism of DIF, MDP enhances the bioavailability of DIF, thereby increasing the toxic effects of DIF. This is consistent with a study by Lasch et al. (2021), which demonstrated increased DIF-mediated effects when DIF was in mixture with fludioxonil, a CYP3A4 inhibitor. Since recombinant CYPs and HLMs lack the complexity of human liver function and xenobiotic metabolism, a more complex system such as HepaRG cell line is necessary to further investigate CYP interactions. Therefore, we evaluated how DIF and MDP affect CYP enzymes at a transcriptional level impacting metabolism, detoxification, and toxicity pathways.

Transcriptomic-derived gene expression profiles enabled us to identify specific CYP genes that are either downregulated or upregulated in response to varying concentrations of DIF and MDP, individually and in mixture.

MDP downregulated the expression of CYPs (Fig. 7), revealing a potential negative impact on the overall activity of all relevant xenobiotic-metabolizing enzymes.

CYP3A4 is the chief metabolizing enzyme of the steatotic active substance DIF. The enzyme was induced at low and inhibited at high concentrations by DIF and DIF + MDP at ratios of 1:1 (equivalent to the formulated product) and 1:8 and the formulated product, as formulated, indicating a biphasic CYP3A4 regulation. CYP3A4 induction by xenobiotics is mainly mediated by a PXR-dependent mechanism that can trigger the transcription of genes encoding CYP3A4 (Hakkola et al. 2020; Luo et al. 2002). The observed induction of CYP3A4 is associated with the triazole-PXR activation as shown in Fig. 7.

Our results are consistent with those of previous studies of triazoles. Similar to DIF, triazoles induce the mRNA of AHR, CAR, and PXR target genes (de Sousa et al. 2014; Knebel et al. 2018; Karaca et al. 2023). Furthermore, a study by Lasch et al. (2021) demonstrated that DIF activates PXR and AHR while downregulating CAR. In contrast to CYP inhibition, induction can occur at low concentrations because nuclear receptor–ligand interactions rely on high sensitivity and downstream amplification. The CYP3A4 relative downregulation demonstrated by high concentrations of DIF and DIF + MDP in ratios of 1:1 and 1:8 and the formulated product could be linked to a PXR negative feedback loop or direct and/or indirect PXR functional interference, which is indicated by downregulation of PXR (Fig. 7). According to Bailey et al. (2011), at high agonist concentrations, PXR negative feedback is used to attenuate PXR-mediated activation of target genes such as CYP3A4. Consequently, this reduces the expression of CYP3A4 at the transcriptional level.

However, while transcriptomics data provide valuable insights on gene activity, it does not quantify protein levels or other cell functional outputs. Therefore, conclusions should be based on a comprehensive evaluation of all relevant data using a weight of evidence approach.

DIF was degraded at a slower rate in the culture medium and cells in the presence of MDP, indicating decreased metabolism of the compound. In the formulated product and formulated product-equivalent mixture (1:1) (Fig. 5), DIF net uptake was decreased, indicating faster degradation as well as toxicokinetic interactions beyond MDP and DIF. In addition to active substances, PPPs contain co-formulants, which are specifically incorporated into formulations to modify the stability, dissolution, and release rate of active substances (Kalyabina et al. 2021). Moreover, surface-active co-formulants have been shown to enhance the bioavailability of active substances by increasing membrane fluidity. This membrane fluidization has been suggested as an underlying mechanism for increased diffusivity and interaction with transporters and metabolic enzymes (Woodcock et al. 1992; Dudeja et al. 1995; Rege et al. 2002; Weinheimer et al. 2016; Karaca et al. 2021). Therefore, it would seem reasonable to assume that, in addition to the inhibition of DIF metabolism by MDP, co-formulants may also contribute to the observed concentration of DIF with respect to the formulated product. This is consistent with previous studies that demonstrated the relevance of co-formulants in PPP kinetics (Zahn et al. 2017; Karaca et al. 2021; Feiertag et al. 2023). In this study, quantification was conducted using only one concentration of DIF alone, its formulated product-equivalent in mixture (1:1) with MDP, and in the formulated product, respectively. Elaborate studies such as time-resolved experiments with multiple test concentrations are required to investigate the contribution of individual co-formulants to MDP and DIF mixture effects.

While the binary active substance mixture with DIF and MDP corresponding to formulated product followed an additive behavior, increasing the concentration of MDP resulted in a more than additive cytotoxic effect, as evidenced by the leftward shift of the modelled curve for at least the higher concentrations of the 1:8 mixture (Fig. 2B). The results indicate that the combination of DIF with an enhancer may increase the concentrations to critical levels where cytotoxicity may occur, even if DIF concentrations are below thresholds that generate toxicity. To support this, the quantity of DIF left in the cell culture medium and HepaRG cells was the highest in the 1:8 mixture compared to DIF alone, the formulated product-equivalent mixture (1:1) and the formulated product (Fig. 5). This is consistent with a previous study, which reported that the concentration of the inhibitor in a mixture determines the extent to which the inhibitor affects the metabolism of a compound (Lynch and Price 2007). Our findings support the conclusion drawn by Bloch et al. (2023) that the toxicological enhancer (MDP) must exceed its effect threshold, which may not cause adverse effects to cause CYP inhibition and generate synergism. Therefore, MDP concentration must be sufficiently high to saturate CYP enzymes and displace DIF at the active site, thereby increasing DIF concentration at the target site and, consequently, higher toxicity.

The findings indicate that toxicokinetic interactions arising from MDP inhibiting DIF-metabolizing CYP enzymes are crucial in the mixture effects observed in the formulated product. However, the concentration of MDP in the formulated product is insufficient to saturate the CYP enzymes and displace DIF at the active site; consequently, the resulting toxicity of the formulated product is not synergistic compared to DIF alone, as evidenced by the EC_50_ values (Table 2). In addition, the toxicokinetic properties of co-formulants may have contributed to the observed mixture effects.

According to in silico prediction and in vitro confirmation, MDP inhibits DIF metabolism via CYP3A4 and CYP2C9, thus the two active substances in the formulated product interact kinetically. Relevant CYP enzyme expression was biphasic, with low concentrations inducing and high concentrations inhibiting CYP3A4 expression. Nonetheless, CA adequately described MDF and DIF cytotoxicity in HepaRG cells at ratios representative of the formulated product. At higher relative concentrations of the enhancer (MDP), more than additive effects were observed, yet they were limited to high effect concentrations. In addition, uptake experiments into HepaRG cells revealed a decreased net uptake in the presence of co-formulants compared to the combination of the active substances alone, thus hinting at antagonistic effects. However, as no time-resolved experiments were conducted and co-formulant toxicity was not investigated, this conclusion remains tentative.

### Toxicodynamic interactions

In addition to kinetic interaction, we also evaluated potential dynamic interaction between DIF and MDP with a focus on liver steatosis.

The formulated product-equivalent mixture (1:1) and the formulated product induced significant modulation of genes related to KEs in the liver steatosis AOP (Mellor et al. 2015; Vinken 2015), beyond what was predicted by individual substance effects, indicating potential toxicodynamic interactions. The toxicodynamic interaction between DIF and MDP in promoting liver steatosis is evidenced by the combined downregulation of both PPARA and ACOX1 (Fig. 7), along with increased triglyceride accumulation (Fig. 6). Individually, DIF and MDP downregulated the expression of PPARA, a critical regulator of lipid metabolism, and subsequently downregulated its target gene, ACOX1, a key enzyme in fatty acid beta-oxidation (Pawlak et al. 2014; Lu et al. 2024). However, the formulated product-equivalent mixture (1:1) and the formulated product exhibit similar downregulation of both PPARA and ACOX1 at concentrations lower than either of the substances alone (Fig. 7). This interaction was further corroborated by AdipoRed assay results (Fig. 6), which revealed a significant increase in intracellular triglyceride accumulation at lower concentrations of the formulated product-equivalent mixture (1:1) and the formulated product. This suggests that the formulated product-equivalent mixture (1:1) and the formulated product disrupts the PPARA-ACOX1 pathway more effectively than individual exposures, potentially through combined impacts on upstream signal pathways or by influencing receptor interactions.

The toxicodynamic interactions of the active substances extend to the de novo fatty acid synthesis pathway, as evidenced by the combined upregulation of MLXIPL and FASN (Fig. 7). Individually, DIF and MDP upregulated the expression of LXR and PPARG (Fig. 7), two nuclear receptors that regulate interconnected pathways involved in lipogenesis (Schadinger et al. 2005; Matsusue et al. 2014). DIF demonstrated LXR-mediated de novo fatty acid synthesis, evidenced by the upregulation of MLXIPL, a glucose-sensitive transcription factor promoting hepatic conversion of excess carbohydrates to lipids (Benhamed et al. 2012; Matsusue et al. 2014). Concurrently, MDP showed a PPARG-mediated de novo fatty acid synthesis pathway, revealing upregulated the expression of fatty acid synthase (FASN), a key enzyme of hepatic de novo lipogenesis converting dietary sugars metabolites into palmitate (Dorn et al. 2010; O’Farrell et al. 2022). Previous studies identified MLXIPL as an LXR target gene, whose activation leads to the stimulation of lipogenic genes, including acetyl-CoA carboxylase and FASN (Benhamed et al. 2012; Matsusue et al. 2014). Furthermore, FASN has been reported as a target for PPARG in addition to other target genes (Chen et al. 2023). Interestingly, the formulated product exhibited upregulation of LXR, PPARG, MLXIPL, and FASN at concentrations lower than either of the substances alone (Fig. 7). These findings indicate toxicodynamic interactions between DIF and MDP, which influence the steatotic effects of the formulated product through distinct yet converging mechanisms that are more effective than individual exposures. This interaction was further corroborated by a significant increase in intracellular triglyceride accumulation at lower concentrations of the formulated product (Fig. 6).

As previously discussed, DIF activates PXR and AHR while downregulating CAR (Chaturvedi et al. 2010; Lasch et al. 2021), whereas MDP activated AHR and downregulated CAR and PXR (Fig. 7). Triazole mRNA induction of AHR, CAR, and PXR target genes has been observed both in vivo and in vitro (de Sousa et al. 2014; Knebel et al. 2018; Karaca et al. 2023). However, the formulated product-equivalent mixture (1:1) and the formulated product downregulated CAR and PXR while upregulating AHR at concentrations lower than those required for individual substances (Fig. 7). This altered NR profile disrupts the balance of xenobiotic metabolism and lipid homeostasis. The observed upregulation of AHR may promote inflammatory pathways and further exacerbate lipid accumulation (Rakateli et al. 2023). Although the established liver steatosis AOP has revealed that upregulation of CAR and PXR triggers KEs that ultimately result in hepatic lipid accumulation (Mellor et al. 2015; Vinken 2015), the interplay between these receptors is complex and varies depending on specific ligands and cellular context, which could explain the exhibited steatotic effect from the formulated product-equivalent mixture (1:1) and the formulated product. These findings are further substantiated by the increased intracellular triglyceride accumulation at the formulated product-equivalent mixture (1:1) and the formulated product concentrations lower than individual substances (Fig. 6).

The in vitro assays demonstrate AOP-aligned liver steatosis disruption, particularly with the formulated product-equivalent mixture (1:1) and the formulated product, raising hepatic steatosis concerns. However, given that CA is the default assumption for mixture risk assessment, it is necessary to assess whether the components contribute to the observed effects via similar or dissimilar MoAs.

The CA approach is a conservative method for cumulative risk assessment for active substances within the same CAG (Backhaus and Faust 2012; More et al. 2019; Braeuning et al. 2022). With respect to PXR activation and its critical role in triazole-induced steatosis (Knebel et al. 2018), it is plausible to assume that DIF and MDP act via dissimilar MoAs, consistent with CAG grouping as MDP is not included in the hepatocellular fatty changes CAG (Nielsen et al. 2012). However, triazoles have been identified as multi-receptor agonists, rather than specific activators of PXR concerning triglyceride accumulation induction (Hester and Nesnow 2007; Tully et al. 2006; Luckert et al. 2018). Furthermore, with respect to ACOX1 antagonism, it can be assumed that MDP and DIF act via similar MoAs. In addition, as demonstrated by the AdipoRed assay (Fig. 6), MDP may be considered steatotic like DIF, as it induced triglyceride accumulation in HepaRG cells. Nevertheless, it is improbable that MDP (EC_1.5-fold_ ≈ 35 µM) will elicit steatotic effects at concentrations typically observed in blood samples, as most pesticides in environmentally exposed humans are generally present in the nM or pM range (Guéniche et al. 2020; Chedik et al. 2017, 2018b). Guéniche et al. (2019) estimated that the human plasma concentrations for most pesticides in environmentally or occupationally exposed individuals range from 1 nM to 0.5 µM. Moreover, given that MDP undergoes extensive metabolism and lacks bioaccumulation potential (EFSA 2012), its concentration in humans may be even lower. Despite the significant overlap in mechanisms with DIF, MDP is not considered steatogenic at physiologically relevant concentrations. Therefore, it seems reasonable to refrain from adding MDP to the CAG. Furthermore, although MDP and DIF are not within the same CAG, CA seems to be suitable to describe the HepaRG toxicity linked to the formulated product (Fig. 2A). Our findings align with the arguments in publications questioning the relevance of distinguishing between similarly and dissimilarly acting mixture components in risk assessment (EFSA 2013; Van Der Ven et al. 2022; Kortenkamp 2023). Kortenkamp (2023) emphasized that such distinction introduces unnecessary complexity without significantly improving the prediction of mixture effects.

Our study indicates that toxicodynamic interactions also contribute to the bioactivity of the formulated product at lower concentrations. In addition, the negligible difference in the EC_1.5-fold_ values between the formulated product-equivalent mixture (1:1) and the formulated product (Table 4) implies that the formulated product-equivalent mixture (1:1) may account for the steatotic potential of the formulated product, suggesting that co-formulants in the formulated product may counterbalance each other.

### Impact on PPP risk assessment

Our study underscores the necessity of considering both toxicokinetic and toxicodynamic interactions to accurately assess mixture toxicity in PPPs. Currently, CAG- and AOP-based grouping approaches are employed for the combined assessment of active substances, focusing on the toxicodynamic properties of pesticides, such as their respective target organs and MoA, while overlooking potential toxicokinetic interactions (Braeuning et al. 2022; Karaca et al. 2023). While both, kinetic and dynamic, interactions were identified between the two active substances in the formulated product, cytotoxicity observations were additive in the PPP-corresponding ratio. This supports the application of Hazard Index to assess the combined toxicity of active substances. Since MDP is not considered a liver steatotic agent according to the CAG system, PPP risk assessment refinement may have resulted in rejecting the similar MoA hypothesis, building the basis for CA and the HI approach (Stein et al. 2014). Our results indicate that additivity is adequate even for substances in different CAGs. As argued above, MDP possesses liver steatotic qualities. Transcriptomics even indicate a dynamic interaction of the MDP and DIF regarding the liver steatosis AOP. Yet, MDP steatotic effects or dynamic interaction with DIF is unlikely to occur at relevant exposure concentrations. In general, where similar MoA can be assumed or kinetic interaction may occur, our results support the application of CA and HI for active substances within PPPs. Further PPPs need to be tested to conclude whether the refinement of the HI approach should be discarded, in general. Furthermore, only when increasing the ratio of the active substance mixture in favor of the synergist (MDP) more than additive effects were observed. However, these were limited to high effect concentrations unlikely to be of human relevance. In such cases, synergistic effects seem improbable as enzyme saturation cannot be achieved. Co-exposure to multiple enzyme inhibitors should be tested in future to confirm this hypothesis beyond single PPP exposure.

In the formulated product, liver toxicity is driven by the two active substances, MDP and DIF. Co-formulants increase neither HepaRG cytotoxicity (Fig. 1), nor triglyceride accumulation (Fig. 6). The cellular net uptake of the toxicological driver substance, DIF, was even decreased in the presence of co-formulants compared to the formulated product-equivalent mixture (1:1) of DIF and MDP (Fig. 5). The simultaneous decrease of DIF in cell medium indicates that this may be caused by increased cellular uptake of DIF in the presence of co-formulants, leading to faster metabolization and thus lower effects.

## Conclusion

As demonstrated in this study, predictive in silico tools such as ADMET predictor® can be utilized to prioritize PPPs containing active substances that may potentially exhibit metabolic-related or transporter-related toxicokinetic interactions in mixtures. Furthermore, this study revealed the significance of both toxicokinetics and toxicodynamics in mixture toxicity. Notably, at lower concentrations, toxicodynamics play a pivotal role, while toxicokinetic interactions enhance more than additive effects in cases where the inhibitor concentration is sufficiently high to saturate enzyme kinetics. Instead of refining the HI approach by disregarding CA for substances, which do not share a target organ, future steps might be directed towards whole mixture risk assessment. Where in vitro points of departure can be arrived at for a mixture of active substances of the whole PPP*,* in vitro–in vivo extrapolation (IVIVE) is a powerful tool to derive human relevant toxicological threshold values. Consequently, in the next step, we will, therefore, investigate strategies to derive PPP-based points of departure and develop an IVIVE strategy for PPPs.

## Supplementary Information

Below is the link to the electronic supplementary material.

Supplementary file1 (XLSX 99 KB)

Supplementary file2 (XLSX 48 KB)

Supplementary file3 (DOCX 153 KB)

## Data Availability

The data that support the findings of this study are available from the corresponding author, Y.M, upon reasonable request. Data on the composition of the PPP is subject to confidentiality and cannot be disclosed. Data on ADMET prediction, cytotoxicity and transcriptomics is provided in the supplementary information to this publication.

## Abbreviations

AhR: Aryl hydrocarbon receptor
AIC: Akaike information criteria
AOP: Adverse outcome pathway
CA: Concentration addition
CAG: Cumulative assessment group
CAR: Constitutive androstane receptor
CYP: Cytochrome P450
DGE: Differential gene expression
DIF: Difenoconazole
DIF d6: Difenoconazole-d6
DMSO: Dimethyl sulfoxide
FCS: Fetal calf serum
FDR: False-discovery rates
IA: Independent action
KEs: Key events
MDP: Mandipropamid
MIE: Molecular initiating event
MoA: Mode of action
MRM: Multiple reaction monitoring
NAMS: New approach methodologies
NR: Nuclear receptors
NRU: Neutral red uptake
PBS: Phosphate-buffered saline
PCA: Principal component analysis
P-gp: P-glycoprotein
PPP: Plant protection product
PXR: Pregnane X receptor
QuEChERS: Quick, Easy, Cheap, Effective, Rugged, and Safe
RIPA: Radioimmunoprecipitation assay
WST-1: Water-soluble tetrazolium
