# Supplementary material for: Relevance of kinetic interactions and co-formulants for plant protection product liver toxicity in vitro
Source: Arch Toxicol. 2025 Apr 28;99(8):3247–68. doi: 10.1007/s00204-025-04071-7 (PMC12367838; doi:10.1007/s00204-025-04071-7)
Supplement: Supplementary file 3 — Supplementary file3 (DOCX 153 KB) [file 204_2025_4071_MOESM3_ESM.docx]

Article name: Relevance of kinetic interactions and co-formulants for plant protection product liver toxicity in vitro

Journal name: Archives of Toxicology

Author names: Yemurai Musengi^1,3^, Ilinca Suciu^1^, Tewes Tralau^2^ and Denise Bloch^1^

Affiliation: ^1^Department of Pesticides Safety, German Federal Institute for Risk Assessment (BfR), Max-Dohrn-Straße 8-10, 10589 Berlin, Germany.

^2^German Federal Institute for Risk Assessment (BfR), Max-Dohrn-Straße 8-10, 10589 Berlin, Germany.

^3^University of Potsdam, Institute of Nutritional Science, Department of Nutritional Toxicology, Arthur-Scheunert-Alle 114-116, 14558 Nuthetal, Germany.

Email address of corresponding author: [Yemurai.Musengi@bfr.bund.de](mailto:Yemurai.Musengi@bfr.bund.de)

Fig S1: Results of WST-1 cytotoxicity test in HepaRG cells after 72 h exposure to increasing concentrations of DIF, MDP, a mixture of DIF and MDP as featured in the formulated product, a 1:8 (DIF:MDP) mixture and the formulated product. The data is presented as percentage of viable cells related to the solvent control. The data represent mean values ± SD of n=3 biological replicates, each performed in three technical replicates. An asterisk (*) indicates statistical significance with α ≤ 0.05 after a linear-mixed-effects ANOVA statistical analysis followed by a post-hoc Dunnet test.

Table S1: EC_50_ values for cytotoxicity derived from best fit values of a non-linear dose-response curve of all treatment substances on HepaRG cells after 72 h incubation.

|  | DIF | MDP | DIF + MDP_1:1 | DIF + MDP_1:8 | Formulated product |
| --- | --- | --- | --- | --- | --- |
| **EC_50_ (mg/L)** | 11.8 | 245 | 6.29 | 3.41 | 9.76 |
| 95% CI | 10.5 to 13.9 | 160 to 676 | 5.62 to ?? | 3.13 to 3.73 | ??? |
